# Spliceosomal intron size expansion in domesticated grapevine (Vitis vinifera)

**DOI:** 10.1186/1756-0500-4-52

**Published:** 2011-03-08

**Authors:** Ke Jiang, Leslie R Goertzen

**Affiliations:** 1 Cold Spring Harbor Laboratory, Cold Spring Harbor, NY, 11724, USA; 2 Department of Biological Sciences, Auburn University, Auburn, AL, 36849, USA

## Abstract

**Background:**

Spliceosomal introns are important components of eukaryotic genes as their structure, sizes and contents reflect the architecture of gene and genomes. Intron size, determined by both neutral evolution, repetitive elements activities and potential functional constraints, varies significantly in eukaryotes, suggesting unique dynamics and evolution in different lineages of eukaryotic organisms. However, the evolution of intron size, is rarely studied. To investigate intron size dynamics in flowering plants, in particular domesticated grapevines, a survey of intron size and content in wine grape (Vitis vinifera Pinot Noir) genes was conducted by assembling and mapping the transcriptome of V. vinifera genes from ESTs to characterize and analyze spliceosomal introns.

**Results:**

Uncommonly large size of spliceosomal intron was observed in *V. vinifera *genome, otherwise inconsistent with overall genome size dynamics when comparing *Arabidopsis*, *Populus *and *Vitis*. In domesticated grapevine, intron size is generally not related to gene function. The composition of enlarged introns in grapevines indicated extensive transposable element (TE) activity within intronic regions. TEs comprise about 80% of the expanded intron space and in particular, recent LTR retrotransposon insertions are enriched in these intronic regions, suggesting an intron size expansion in the lineage leading to domesticated grapevine, instead of size contractions in *Arabidopsis *and *Populus*. Comparative analysis of selected intronic regions in V. vinifera cultivars and wild grapevine species revealed that accelerated TE activity was associated with grapevine domestication, and in some cases with the development of specific cultivars.

**Conclusions:**

In this study, we showed intron size expansion driven by TE activities in domesticated grapevines, likely a result of long-term vegetative propagation and intensive human care, which simultaneously promote TE proliferation and repress TE removal mechanisms such as recombination. The intron size expansion observed in domesticated grapevines provided an example of rapid plant genome evolution in response to artificial selection and propagation, and may shed light on the important genomic changes during domestication. In addition, the transcriptome approach used to gather intron size data significantly improved annotations of the V. vinifera genome.

## Background

Eukaryotic genes contain spliceosomal introns that are post-transcriptionally removed by the spliceosome, an RNA-protein complex [1]. The length, position and phase of spliceosomal introns are important components to the evolution of genome architecture [2]. However, most intronic regions are often considered 'junk' DNA similar to intergenic regions or other non-coding sequences. Increasing scrutiny of genomic data has revealed many conserved non-coding sequences (CNS) in the non-coding DNA of both plant and animal genomes [3]. CNS may conduct important functions and experience selective constraints [4]. One major function of CNS is regulating gene expression via interactions between small DNA motifs and transcription machinery (transcription factors and RNA polymerase) [5]. The regulatory motifs are enriched in 5' promoter regions and the first introns of plant and animal genes [6].

Plant genomes usually have compact genes due to small introns [7]. Arabidopsis, the plant model system first completely sequenced, has an average gene size of 2000 bp and average intron size of 180 bp [8]. Intron size is hypothesized to be constrained by energy use in transcription, as large introns may require more energy to be transcribed and spliced, so there is selection against introns of excessive size [9]. However, in certain genes, selection against intron size may be counteracted by a selective preference for bigger introns with more regulatory elements and finer control of gene expression [10].

Transposable elements (TEs) are known to be major components of plant genomes [11]. The origin and proliferation of TEs in plant genomes shaped plant genome dynamics and genetic diversity. Unlike the human genome in which TEs are predominantly in introns [12], plant TEs are usually found in intergenic regions, possibly a result of strong purifying selection against TE insertions in exons and introns [13]. The observed plant genome size expansions of cereals are thought to be results of whole-genome duplications and TE invasions in non-coding regions [14]. However, in a few cases, TE insertions in introns altered temporal and spatial expression patterns of specific genes. The insertions caused significant genetic and phenotypic changes and seemed to be preserved by natural selection [15]. During plant domestication, key traits selected by human may be the result of genetic diversity generated by TE insertions [16].

To further investigate intron size evolution in plant genomes, we present a novel approach to identify and analyze introns of large size using genomic data for a domesticated grapevine cultivar (Vitis vinifera Pinot Noir). The specific objectives are to investigate the size distribution of introns; relationship between intron size and gene function, determine the contents of introns and examine the associations between intron size evolution and grapevine domestication. Generally, we show that introns of truly extraordinary size are widespread in cultivated grapevines, a phenomenon not observed in other plant genomes.

## Results

### EST collection, processing and assembly

In total, 353,748 publicly available EST sequences of V. vinifera were clustered into 26,111 clusters, 8,560 of which represented by only one EST sequence. The largest cluster is composed of 1,998 EST sequences. The distribution of cluster size suggested that one-third of V. vinifera genes are not expressed at very high levels (32.8% singletons) and a few (3 clusters contain more than 1000 ESTs) genes are highly expressed across the representative EST libraries. Among the 26,111 clusters, 5,678 clusters contain no intron, while 20,433 clusters were predicted to be spliced. Among all clusters, 17,059 clusters were predicted to encode proteins, in which 15,369 predicted proteins show homology to *Arabidopsis *proteins and 15,683 proteins show homology to *Populus *proteins (BLASTP, E-value < 0.001). BLAST searches against an EST database of other Vitis species showed that 15,089 clusters (60.5%) had high similarity hits in at least one other species of grapevine.

### Identification and annotation of large genes and introns

The average intron size, predicted by the annotation of the first draft sequences of *Vitis vinifera *Pinot Noir, is slightly larger than other sequenced plants [17]. After mapping the assembled consensus sequences to V. vinifera genomic regions, 2,697 introns having size between 3 kb and 100 kb were identified in 2,563 genes (10% of all assembled clusters, 13% of spliced clusters). Among these genes, 1,179 (46.0%) only have one EST sequence (singletons), a higher proportion than among all predicted genes from EST clusters (32.8%). This indicates that if the number of EST sequences in cluster is a good indictor of expression level, genes with large introns had lower expression level compared to all genes (46.0% vs 32.8% singletons).

**Figure 1:**
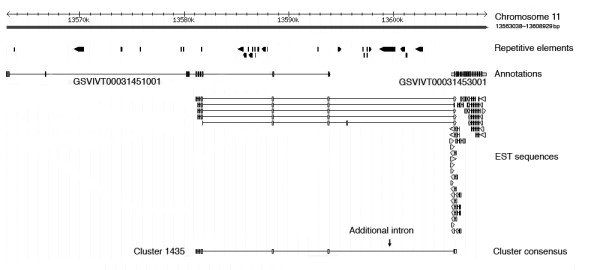
**Correction of annotation error due to large introns**. Additional intron needed for correct annotation of Vitis vinifera gene by comparing EST sequences, cluster consensus sequence and known annotation. The diagram shows the comparison between genomic sequences (Chromosome 11, 13563038 to 13608929 bp), known annotation (GSVIVT00031451001 and GSVIVT00031453001) and gene expression data (ESTs and cluster 1435). Crossed bars, genomic sequences with coordinates; black bars with arrows, repetitive elements; filled boxes (some with arrows), exons; lines, introns. Additional intron needed indicated by small arrow on top of cluster consensus sequence.

Comparisons between the 2563 clusters (predicted genes from EST clusters) and existing gene annotations of the V. vinifera genome revealed that 1,812 clusters matched to known annotations with 100% identity, with 1,137 matches that showing identical transcription start/end sites and exon/intron structure between our predictions and known annotations. However, in 217 matches, multiple EST-predicted genes (487 clusters) matched to single annotated genes, while 188 clusters matched to multiple annotated genes. The later case represented one type of annotation error in V. vinifera genome, in which a single gene supported by expression data was incorrectly split into multiple annotated genes due to a large intron between coding regions. Among the 188 clusters, 99 clusters correspond to multiple, consecutively annotated genes, suggesting that additional introns are needed between annotated genes to correct the annotations (Figure 1).

**Figure 2:**
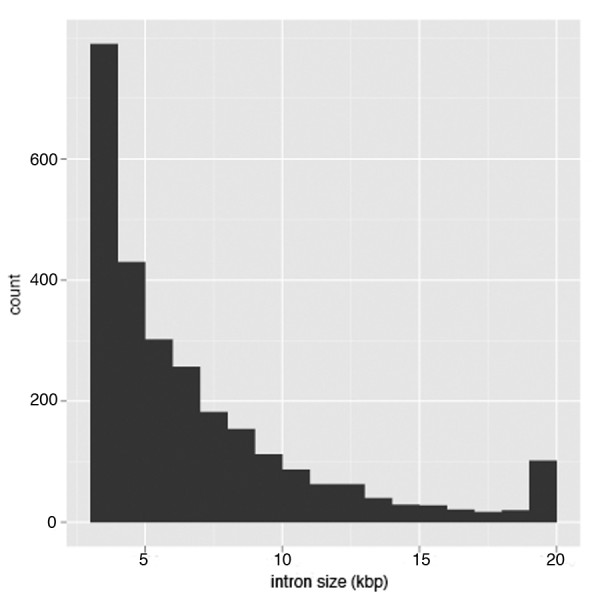
**Large intron size distribution**. Size distribution of large introns identified in V. vinifera (> 3000 bp). Last bar on the right represent all introns larger than 20 kb.

**Figure 3:**
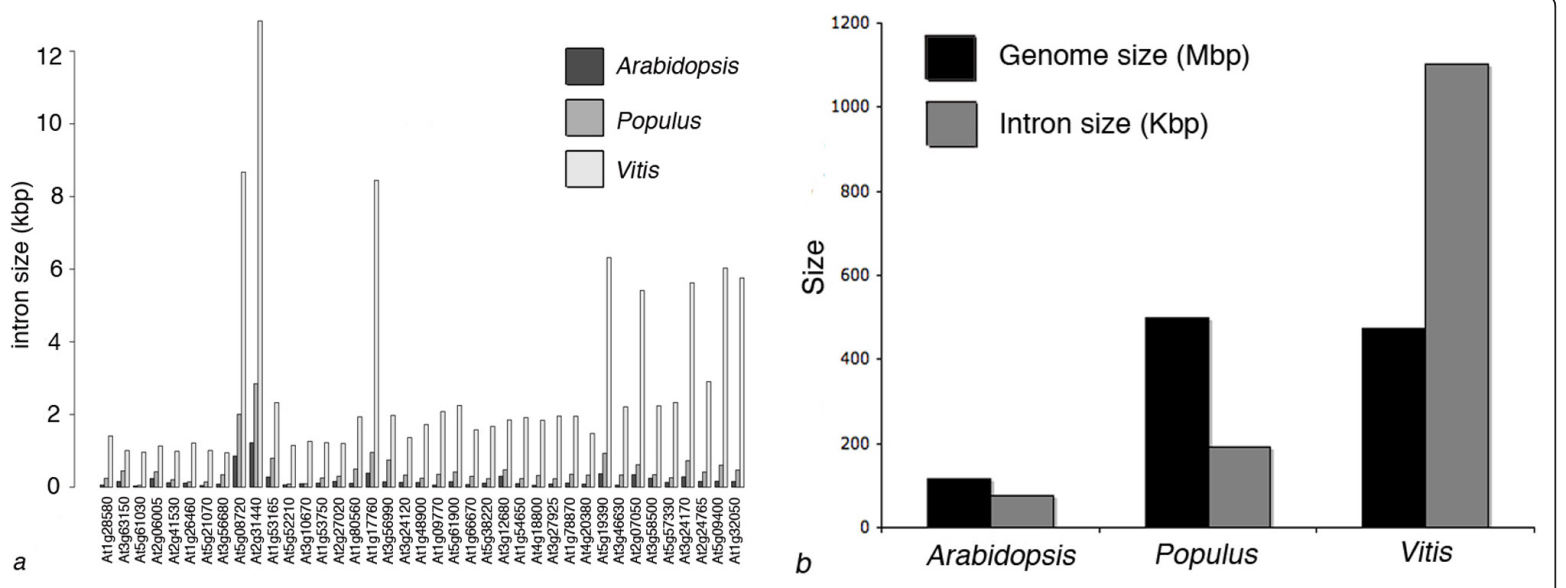
**Intron and genome size comparisons among three plants**. Intron and genome size comparisons among Arabidopsis, Populus and Vitis: a. Overall intron size (kbp) and genome size (mbp) comparison; b. Intron size variation at gene-by-gene base

The function or gene ontology of the 2,563 genes, suggested by their *Arabidopsis *and *Populus *homologs and Pfam protein domain families, does not show bias to any particular gene family, indicating that intron size is not correlated with gene function. In the 2,563 genes, 2,697 introns between 3 kb to 100 kb (cf. 20 in Arabidopsis and 54 in Populus, respectively) were identified (Figure 2). Among Arabidopsis, Populus and Vitis, genes with conserved exon/intron structure, which means identical exons/intron numbers, intron positions and phase, were identified for detailed intron size comparisons. Because *Vitis *genome is still in an intensive annotation process, causing constant modifications of annotation, UTR regions are not considered in this study. Only 39 genes having conserved exon/intron structure in Arabidopsis and Populus were identified from the 2,563 genes and subjected to one-to-one comparison, which will be an underestimate because of the uncertainties in *Vitis *annotation. This is also supported by the fact that a lot of remaining genes showing an extra or missing exon at 3' or 5' end in *Vitis*. Among the 39 genes, 74 introns larger than 3 kb were identified. The one-to-one comparison of intron sizes revealed that the V. vinifera genome, four times larger than Arabidopsis overall, has experienced a 12-fold expansion of intron size in these 39 genes. The genome size of V. vinifera is nearly identical to Populus but experienced a 5-fold expansion of intron size in these 39 genes (Figure 3b). The increase in intron size is generally the same in each individual gene (Figure 3a). In addition, large introns in the 39 genes are predominantly 5'-introns (over 40% intron 1 and intron 2).

### Contents of large introns and TEs

**Figure 4:**
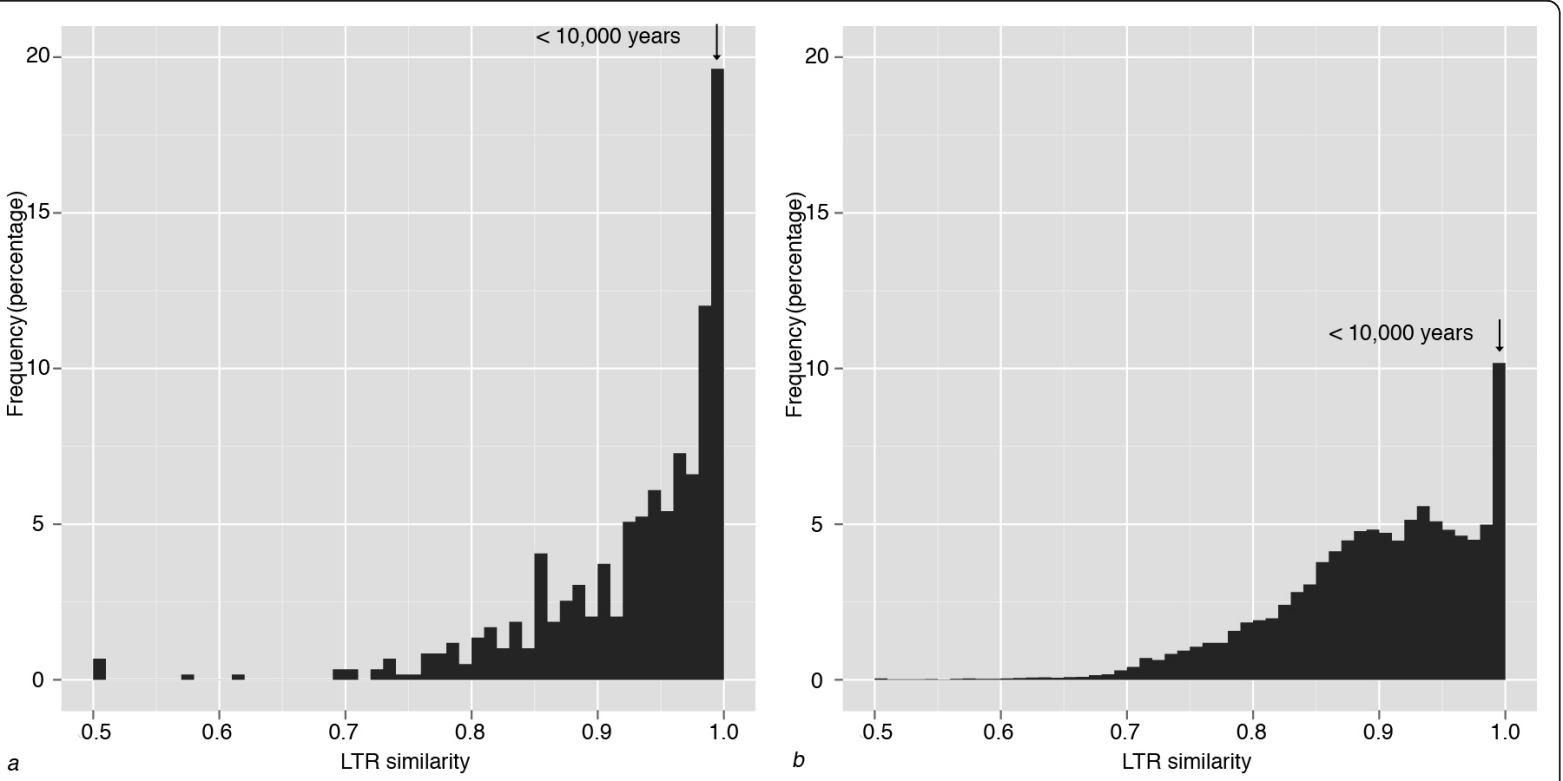
**Age distributions of LTR-retrotransposons in introns and genome**. Distribution of LTR retrotransposon age represented by similarity between LTR regions (the higher similarity, the younger the elements) in a. large introns; b. V. vinifera genome. LTR pairs younger than 10,000 years are indicated by arrows

RepeatMasker screening and BLAST searches of the 2,697 large introns suggested that over 80% of the intron contents were made up of repetitive elements. The dominant types of repetitive elements are low complexity sequences, simple repeats, LTR retrotransposons and LINEs. The number of Copia-type LTR retrotransposons is about 6.5 times that of the Gypsy-type, in contrast to less Copia than Gypsy in the V. vinifera genome overall [17]. The distribution of similarities between flanking LTR regions of the 591 complete LTR retrotransposons is shown in Figure 4a. When compared to the similarities between LTR regions identified in V. vinifera genome overall (Figure 4b), LTR regions in introns contain more highly similar pairs (about 30% of the pairs with identity higher than 99%, |^2^, P < 0.01), suggesting younger LTR retrotransposon insertions and recent LTR retrotransposition activity in these introns. Based on an estimate of substitution rate in LTR regions, age estimates of LTR retrotransposons in introns identified a burst of LTR retrotransposon activities: <10,000 ya (LTR similarity 100%) (Figure 4a). For these young LTR-retrotransposons, Copia/Gypsy ratio is 5.8, close to the ratio for all LTR-retrotransposons in large introns, indicating that recent LTR-retrotransposon proliferation is not biased towards either retrotransposon family.

In the 39 genes with identical exon/intron structure/number in Arabidopsis and Populus, repetitive elements comprised roughly 90% of the 74 intronic regions, in which 10 complete LTR retrotransposons were identified. Among the 74 introns, 1 intron contains only a LINE; 1 intron contains only LTR elements; 15 introns contain LINEs and other repetitive elements; 15 introns contain LTR elements and other repetitive elements; 5 introns contain LTR, LINEs and other repetitive elements; 34 introns contain only other repetitive elements; and 3 introns do not contain repetitive elements. According to a BLAST search against the V. vinifera genome, 'other repetitive elements' were highly repetitive within V. vinifera and likely represented un-classified or unnamed grapevine-specific TEs. The three introns without any identified repetitive elements all contain LINE-like ORFs otherwise highly repetitive in the V. vinifera genome.

**Figure 5:**
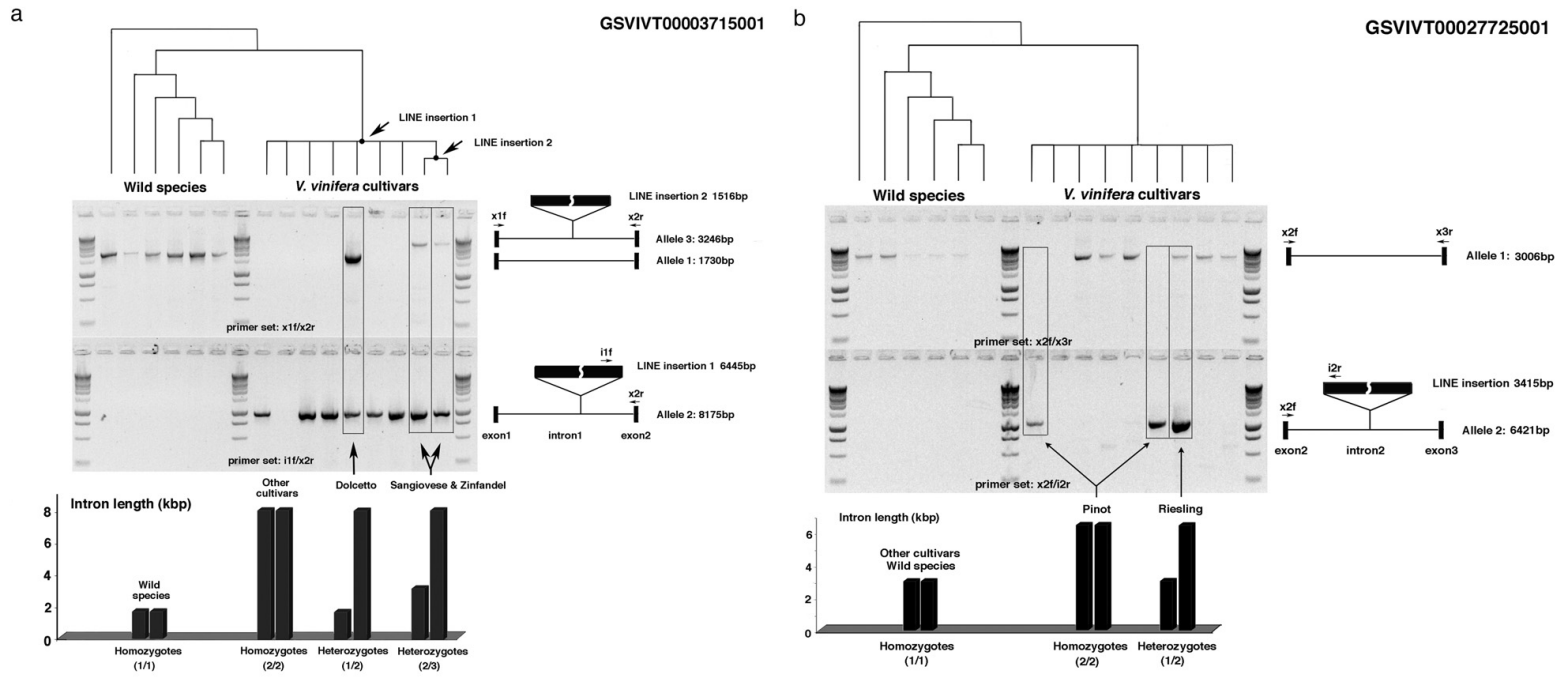
**Characterization of selected introns in grapevine species and varieties**. Intron length variations due to LINE insertions in Vitis species and Vitis vinifera varieties. Phylogenetic tree shows relationships among taxa sampled. Gel picture shows amplification results of alleles shown on the right with primer names LINE insertion size and total allele sizes. Bar graph shows intron size variations among Vitis species and Vitis vinifera varieties including zygotic status. a. GSVIVT0003715001 intron 2; b. GSVIVT00027725001 intron.

Characterizations of four selected introns (all containing LINE insertions) in Vitis species and domesticated varieties revealed intron length variation across these species and varieties (Table 1). None of the native Vitis species have LINE insertions in introns. For the first two introns, the size expansion was not found in any Vitis species or variety, including Pinot Noir, possibly suggesting that the LINE insertion is only present in the specific individual or lineage that was used for genome sequencing. The third intron contained identical LINE insertions in both alleles of Pinot Noir (homozygous), but was heterozygous in Dolcetto (Figure 5a). The fourth intron had LINE insertions in all varieties of domesticated grapevines, but was absent from introns of wild species. Pinot Noir, as well as Cabernet Sauvignon, Chardonnay and Riesling, are homozygous for this LINE insertion, but Dolcetto is heterozygous with one insertion-negative and one insertion-positive allele. In addition, Sangiovese and Zinfandel contain an additional LINE insertion in one allele. Together with a 'Pinot Noir' allele, both varieties are heterozygous for the intron length and carrying additional TE insertions not apparent in Pinot Noir genomic sequences (Figure 5b).

**Table 1:** Characterization of selected introns in wild grapevine species and V. vinifera cultivars

Gene name		GSVIVT00033984001	GSVIVT00022278001	GSVIVT00027725001	GSVIVT0003715001
**Gene functions**		**nucleotide binding**	**signal transduction**	**ATP binding**	**hydrolase activity**

**Intron**		**4**	**12**	**2**	**1**

	*V. rotundifolia*	-/-	-/-	-/-	-/-
	*V. californica*	-/-	-/-	-/-	-/-
Wild Species	*V. girdiana*	-/-	-/-	-/-	-/-
	*V. aestivalis*	-/-	-/-	-/-	-/-
	*V. labrusca*	-/-	-/-	-/-	-/-
	*V. jacquemontii*	-/-	-/-	-/-	-/-

	Cabernet Sauvignon	-/-	-/-	-/-	P2/P2
	Chardonnay	-/-	-/-	-/-	P2/P2
	Dolcetto	-/-	-/-	P1/-	P2/-
*V. vinifera*	Pinot Noir	-/- ^a^	-/- ^a^	P1/P1^b^	P2/P2^c^
	Riesling	-/-	-/-	-/-	P2/P2
	Sangiovese	-/-	-/-	-/-	P2/SZ^d^
	Zinfandel	-/-	-/-	-/-	P2/SZ

Gene names and functions follow Genoscope terminology and annotations [17]. Dashes (-) represent wild-type alleles. Letters (P1, P2, SZ) represent alleles with TE insertions, discovered by genomic analyses. a. Wild-type alleles according to PCR, TE inserted alleles according to genomic sequences, may be unique to the sequenced individual of Genoscope. b. Pinot Noir allele according to both PCR and genomic sequences (P1: 6421 bp). c. Pinot noir allele according to both PCR and genomic sequences (P2: 8175 bp). d. Sangiovese and Zinfandel alleles according to PCR, not found in genomic sequences (SZ: 3246 bp)

## Discussion

### Transcriptome and intron identification

Genome-wide intron analysis has only been done in very few plants [18]. In this study, we identified introns on a whole genome scale by combining a large EST data set and genomic sequences in an automated workflow. This approach has been rarely used before because sufficient EST and genomic data are needed but available for only a few plant species [19]. Domesticated grapevine ranks in the top 25 taxa with the largest collections of ESTs and is the fourth whole plant genome sequence available, making it ideal for the EST mapping approach described here. EST cluster number in this study is higher than the predicted gene number of V. vinifera [17], suggesting that gene prediction based on EST consensus sequences covered a gene range comparable to genomic annotation. By manually checking selected matches between EST consensus sequences and genomic regions, the rate of correct mapping is 100%. Therefore, our workflow accurately identified the corresponding genomic regions of EST consensus sequences, thus correct intron positions and sizes.

Annotation errors such as splitting single gene into multiple ones as a result of unsually long introns, were revealed by comparing ESTs to current annotations (Figure 1). This was expected in two ways: correct prediction of genes in genomic sequences is very sensitive to gene size because large size genes are more likely to be predicted incorrectly due to fragmentation [20]; the large introns present in these genes tend to cause prediction algorithms to split exons at both ends of large introns into two separate genes. Incorporating ESTs into gene prediction algorithms can clearly refine genome annotation as suggested by Coyne et al. [21].

### Properties of genes with large introns

Large size introns are expected to require more energy to be transcribed and spliced, so large introns may decrease gene expression (energy cost hypothesis) [9]. Such hypothesis was supported by this study if we consider that the number of ESTs is a good indicator of gene expression level. However, there is evidence against this hypothesis in genes expressed in specific mammal organs/tissues [22] and plant genomes [23].

In contrast, gene size and/or intron size seems to be not related to gene function in this study. Among identified large genes, there is no particularly overrepresented gene family. There are still about 20% of expanded intronic regions not accounted for by similarity searches or repeat masking, suggesting unique sequences in introns are also evolving. Most of these unique sequences may be evolving neutrally since there may not be any selective constraint on them. However, a few of them may contain very important regulatory elements controlling gene expression patterns thus under selective constraints.

### Intron size expansion, TEs, grapevine evolution and domestication

Comparisons of genome sizes and intron sizes between Populus and Vitis revealed that intron sizes has increased much more than genome size. Either grapevine specific intron expansion or *Populus *specific intron contraction can explain the intron size difference between the two plants. The young LTR retrotransposons and unique presence of these TEs in domesticated grapevine both suggested that an intron size expansion is more likely to be the case. Wendel et al. [18,24] proposed that intron size dynamics may be decoupled with genome size evolution by showing that intron size stayed static in multiple rounds of genome expansion and contraction in cotton (Gossypium). In contrast, we showed that Vitis intron size has experienced a 5-fold expansion compared to Populus, with either unknown fluctuation in genome size, or none at all. Intron size dynamics in Vitis seem to be decoupled from genome size evolution but our data suggested expansion instead of conservation of intron size.

A major force behind the intron size expansion in grapevine is the proliferation of TEs, consistent with previous observations [17]. Among various TEs, LTR retrotransposons provides a proxy to estimate temporal aspects of their activities due to their unique structure. Several studies have estimated the age of LTR retrotransposon insertions in plant genomes such as rice, Medicago and maize [25-27]. Peaks of LTR retrotransposon activity were identified including some that were very recent and possibly related to plant domestication [28,29]. Vitte and Panaud [30] suggested that plant LTR retrotransposons evolved following a 'burst and contraction' model, which could explain the observed 'peaks' of LTR retrotransposon activities in various plant genomes. Different age estimates of Vitaceae family all placed its origin at around 110 million years ago [31-33]. As a result, LTR retrotransposon bursts observed in the introns are much younger than the divergence of Vitales (with a single family Vitaceae) from the common ancestor with rosids, suggesting that increased retrotransposon activity, as well as intron size expansion, is also associated with unique evolutionary changes along the grapevine lineage such as recent domestication.

*V. vinifera *expanded introns contain significantly more identical LTR pairs, suggesting that more young LTR retrotransposons have been preserved in introns of domesticated grapevine (Figure 4a). Here we suggest that the most recent burst of LTR retrotransposons in grapevine may be associated with domestication, either as a result of artificial selection on the nearby genes or genomic regions (local effect), or a non-adaptive by-product of the domestication bottleneck and specific reproduction modes in domesticated grapevines (genome-wide effect). There is no enrichment of any particular gene families in genes with TE invaded introns. Threrfore it is very unlikely that all of the TEs and introns, or nearby genes with diverse functions, which are randomly distributed in genome, were selected by humans in the domestication process. Instead, LTR retrotransposons in the most recent burst were more likely to be preserved in introns than previous bursts due to vegetative clonal propagation of domesticated grapevine plants. Recent and variety-specific TE insertions in introns were discovered in our characterization of large introns from several commonly cultivated grapevine varieties, suggesting that initial TE invasion of introns spread with clonal propagations (see results, Table 1) [34]. This may be explained by the following reasons: first, somaclonal propagation of plants (or tissue culture) was suggested to promote or induce LTR retrotransposon activity [35,36]. Domesticated plants with vegetative propagation may generate more LTR retrotransposons than wild species and non-clonal cultivated plants. Second, plant genome expansions caused by TE proliferation are counteracted by rapid DNA removal as a result of recombination [37]. However, due to a domestication bottleneck and vegetative propagation, the linkage disequilibrium (LD) in cultivated grapevines can be found at extremely long distance compared to wild species [38], suggesting that recombination is repressed in large chunks of chromosomes. TEs, in particular LTR retrotransposons, in domesticated grapevines, may not be removed as effectively as in wild species. Third, LTR retrotransposons, as mutagenesis factors, may cause deleterious mutations if inserted in genes. The mutants were selected against and removed from populations very quickly in natural environments [39]. In domesticated plants, intensive human care produces an environment with relaxed selective constraints, in which LTR retrotransposons invaded genes were more likely to persist. Particularly, vegetatively propagated cultivars such as domesticated grapevine have a uniform genetic background as a result of clonal reproduction to preserve desired traits. This reproduction mode facilitated the spread and fixation of introns with LTR retrotransposons (and other TEs) to the entire clonal population or variety. This may be the major reason why excessive TEs were not observed within genes of other plant genomes, including domesticated plants without clonal propagation such as rice [40,41].

## Conclusions

In this study, the intron size dynamics in domesticated grapevine suggested that intron size expansion, mainly caused by TE invasions, was associated with the plant's unique and recent evolutionary history. Intron size expansion and burst of TE activity may all be related to major evolutionary changes associated with domestication in grapevine. Investigation of intron size expansion not only reveals a unique pattern of plant genome dynamics, but also raises an interesting question: what is the role of introns, a somewhat neglected portion of the genome, in plant genome architecture, function and evolution?

## Methods

### EST data collection, processing and assembly

All currently available V. vinifera Expressed Sequence Tags (ESTs) were obtained from NCBI GenBank using the PartiGene package [42]. Only *V. vinifera *ESTs were included in this study, including over 140 varieties and cultivars. Vector and poly-A sequences were trimmed by the built-in functions of PartiGene. Cleaned EST sequences were clustered using both CLOBB (the default clustering method of PartiGene [43]) and TGICL [44]. Both methods employ megaBLAST [45] searches to produce clusters based on sequence similarity. For both methods, a similarity cut-off of 99% and greater than 100 bp overlap were employed. Both approaches yielded similar numbers of EST clusters and the clustering results of TGICL were adopted for further analyses. The clusters were assembled and the consensus sequences for each cluster were predicted by PHRAP [46]. The protein coding potential of the clusters were predicted by ESTScan [47].

### Identification of large genes and introns

EST consensus sequences were mapped to genomic sequences of V. vinifera Pinot Noir [17] to identify genomic locations and exon/intron structures of the predicted genes. The mapping process was performed with BLAT, by which ESTs or cDNAs were aligned to genomic regions with near identity [48]. Most alignment gaps in BLAT results represent intronic regions. Genes with putative large introns were selected by two criteria: first, the similarity score from BLAT search is higher than 99%; second, the mapped genomic region (excluding introns) cover 95% of the EST consensus sequence. These criteria ensure that predicted gene sequences were mapped to the correct genomic positions. The process was automated with a Perl script for parsing BLAT results, in which the gap sizes were calculated and gap sequences (size range 3 kb~100 kb) and coding regions associated with them were extracted. Extracted gap regions were manually inspected for splicing signals. Gaps with canonical donor and acceptor splice sites (5'-end GT and 3'-end AG) were considered introns. The entire process was also conducted on Arabidopsis and Populus ESTs and genomes to characterize large introns in those plant species.

Genes with putative large introns were submitted to the following analysis: 1, the coding regions were predicted and translated into protein sequences by ESTScan [47]; 2, predicted coding regions were compared to Genoscope annotations by similarity searches and manual corrections; 3, BLAST searches were conducted using the predicted coding regions as queries against an EST database of Vitis species other than V. vinifera. Presence of high similarity hits in non-V. vinifera EST database suggested that the genes have EST data in at least two closely related species, thus they are very likely truly expressed; 4. BLAST searches using predicted protein sequences were conducted against the Arabidopsis and Populus genomic databases to identify the homologous genes in the two plant genomes as an indicator of possible function. In addition, the predicted *V. vinifera *proteins were subjected to HMM search of protein functional domains against Pfam database [49].

### Analysis of large intron contents and selected individual introns

Identified large introns (3 kb~100 kb-long) were subjected to the following analysis: 1, the intronic sequences were screened by RepeatMasker [50] to identify repetitive elements using an Arabidopsis repetitive element library; 2, the introns without any repetitive elements detectable by RepeatMasker were subjected to BLAST searches against the V. vinifera genome. Presence of large number of high scoring hits (cut-off E-value = 0.001) suggests grapevine-specific repetitive elements. 3. Full length LTR retrotransposons in intronic regions were identified by LTR_FINDER [51]. The sequence similarity between two LTR regions were used to estimate the age of LTR retrotransposon insertions using the methods described by SanMiguel et al. [52], with the JC substitution model used to correct for multiple substitutions in LTR regions [53]. All statistical analyses were conducted with the statistical package R [54].

To characterize homologous introns in other Vitis species and varieties, intronic regions that met the following criteria were chosen: 1, there is only one repetitive element in the intron, suggesting a relatively recent expansion; 2, the total intron size without repetitive elements does not exceed 3 kb (to facilitate PCR amplifications). Primers were designed for the flanking exon sequence as well as conserved regions within the repetitive elements to allow scoring either the presence or absence of the elements in a given intron (primer sequences available upon request). PCR amplifications of selected intronic regions were performed for one individual each of six wild grapevine species: V. rotundifolia, V. californica, V. girdiana, V. aestivalis and V. labrusca, V. jacquemontii, and seven V. vinifera cultivars: Cabernert Sauvignon, Chardonnay, Dolcetto, Pinot Noir, Riesling, Sangiovese and Zinfandel. Alleles with or without TE insertions were determined by the size of the PCR product.

## Competing interests

The authors declare that they have no competing interests.

## Authors' contributions

KJ and LRG conceived the study, designed and carried out the experiments, analyzed data, and wrote the manuscript. All authors read and approved the final manuscript.

## References

[B1] Chow L Gelinas R Broker T Roberts R An amazing sequence arrangement at the 5' ends of adenovirus 2 messenger RNA Cell 1977 12 1 1 8 10.1016/0092-8674(77)90180-5 902310

[B2] Irimia M Roy S Spliceosomal introns as tools for genomic and evolutionary analysis Nucleic Acids Res 2008 36 5 1703 12 10.1093/nar/gkn012 18263615 PMC2275149

[B3] Elgar G Pan-vertebrate conserved non-coding sequences associated with developmental regulation Brief Funct Genomic Proteomic 2009 8 4 256 65 10.1093/bfgp/elp033 19752044

[B4] Keightley P Gaffney D Functional constraints and frequency of deleterious mutations in noncoding DNA of rodents Proc Natl Acad Sci USA 2003 100 13402 13406 10.1073/pnas.2233252100 14597721 PMC263826

[B5] Woolfe A Goodson M Goode DK Snell P McEwen GK Vavouri T Smith SF North P Callaway H Kelly K Walter K Abnizova I Gilks W Edwards YJ Cooke JE Elgar G Highly conserved non-coding sequences are associated with vertebrate development PLoS Biol 2005 3 1 e7 10.1371/journal.pbio.0030007 15630479 PMC526512

[B6] Majewski J Ott J Distribution and characterization of regulatory elements in the human genome Genome Res 2002 12 1827 1836 10.1101/gr.606402 12466286 PMC187578

[B7] Hong X Scofield D Lynch M Intron size, abundance, and distribution within untranslated regions of genes Mol Biol Evol 2006 23 12 2392 404 10.1093/molbev/msl111 16980575

[B8] Arabidopsis Genome Initiative Analysis of the genome sequence of the flowering plant Arabidopsis thaliana Nature 2000 408 6814 796 815 10.1038/35048692 11130711

[B9] Castillo-Davis C Mekhedov S Hartl D Koonin E Kondrashov F Selection for short introns in highly expressed genes Nat Genet 2002 31 4 415 8 12134150 10.1038/ng940

[B10] Marais G Nouvellet P Keightley P Charlesworth B Intron size and exon evolution in Drosophila Genetics 2005 170 1 481 5 10.1534/genetics.104.037333 15781704 PMC1449718

[B11] Kumar A Bennetzen J Plant retrotransposons Annu Rev Genet 1999 33 479 532 10.1146/annurev.genet.33.1.479 10690416

[B12] Wong G Passey D Yu J Most of the human genome is transcribed Genome Res 2001 11 12 1975 7 10.1101/gr.202401 11731486

[B13] Bennetzen J Transposable element contributions to plant gene and genome evolution Plant Molecular Biology 2000 42 1 251 69 10.1023/A:1006344508454 10688140

[B14] Messing J Bennetzen J Grass genome structure and evolution Genome Dyn 2008 4 41 56 full_text 18756076 10.1159/000126005

[B15] Lempe J Balasubramanian S Sureshkumar S Singh A Schmid M Weigel D Diversity of flowering responses in wild Arabidopsis thaliana strains PLoS Genet 2005 1 1 109 18 10.1371/journal.pgen.0010006 16103920 PMC1183525

[B16] This P Lacombe T Cadle-Davidson M Owens C Wine grape (Vitis vinifera L.) color associates with allelic variation in the domestication gene VvmybA1 Theor Appl Genet 2007 114 4 723 30 10.1007/s00122-006-0472-2 17221259

[B17] Jaillon O Aury JM Noel B Policriti A Clepet C Casagrande A Choisne N Aubourg S Vitulo N Jubin C Vezzi A Legeai F Hugueney P Dasilva C Horner D Mica E Jublot D Poulain J Bruyère C Billault A Segurens B Gouyvenoux M Ugarte E Cattonaro F Anthouard V Vico V Del Fabbro C Alaux M Di Gaspero G Dumas V Felice N Paillard S Juman I Moroldo M Scalabrin S Canaguier A Le Clainche I Malacrida G Durand E Pesole G Laucou V Chatelet P Merdinoglu D Delledonne M Pezzotti M Lecharny A Scarpelli C Artiguenave F Pè ME Valle G Morgante M Caboche M Adam-Blondon AF Weissenbach J Quétier F Wincker P French-Italian Public Consortium for Grapevine Genome Characterization The grapevine genome sequence suggests ancestral hexaploidization in major angiosperm phyla Nature 2007 449 7161 463 7 10.1038/nature06148 17721507

[B18] Wendel J Cronn R Alvarez I Liu B Small R Senchina D Intron size and genome size in plants Mol Biol Evol 2002 12 2346 52 10.1093/oxfordjournals.molbev.a004062 12446829

[B19] Lanier W Moustafa A Bhattacharya D Comeron J EST analysis of Ostreococcus lucimarinus, the most compact eukaryotic genome, shows an excess of introns in highly expressed genes PLoS One 2008 3 5 e2171 10.1371/journal.pone.0002171 18478122 PMC2367439

[B20] Wang J Li S Zhang Y Zheng H Xu Z Ye J Yu J Wong G Vertebrate gene predictions and the problem of large genes Nat Rev Genet 2003 9 741 9 10.1038/nrg1160 12951575

[B21] Coyne RS Thiagarajan M Jones KM Wortman JR Tallon LJ Haas BJ Cassidy-Hanley DM Wiley EA Smith JJ Collins K Lee SR Couvillion MT Liu Y Garg J Pearlman RE Hamilton EP Orias E Eisen JA Methé BA Refined annotation and assembly of the Tetrahymena thermophila genome sequence through EST analysis, comparative genomic hybridization, and targeted gap closure BMC Genomics 2008 9 562 10.1186/1471-2164-9-562 19036158 PMC2612030

[B22] Huang Y Niu D Evidence against the energetic cost hypothesis for the short introns in highly expressed genes BMC Evol Biol 2008 8 154 10.1186/1471-2148-8-154 18492248 PMC2424036

[B23] Ren XY Vorst O Fiers MW Stiekema WJ Nap JP In plants, highly expressed genes are the least compact Trends Genet 2006 22 10 528 32 10.1016/j.tig.2006.08.008 16934358

[B24] Wendel J Cronn R Johnston J Price H Feast and famine in plant genomes Genetica 2002 115 1 37 47 10.1023/A:1016020030189 12188047

[B25] Vitte C Panaud O Quesneville H LTR retrotransposons in rice (Oryza sativa, L.): recent burst amplifications followed by rapid DNA loss BMC Genomics 2007 8 218 10.1186/1471-2164-8-218 17617907 PMC1940013

[B26] Wang H Liu J LTR retrotransposon landscape in Medicago truncatula: more rapid removal than in rice BMC Genomics 2008 9 382 10.1186/1471-2164-9-382 18691433 PMC2533021

[B27] SanMiguel P Tikhonov A Jin YK Motchoulskaia N Zakharov D Melake-Berhan A Springer PS Edwards KJ Lee M Avramova Z Bennetzen JL Nested retrotransposons in the intergenic regions of the maize genome Science 1996 274 5288 765 8 10.1126/science.274.5288.765 8864112

[B28] Moisy C Garrison K Meredith C Pelsy F Characterization of ten novel Ty1/copia-like retrotransposon families of the grapevine genome BMC Genomics 2008 9 469 10.1186/1471-2164-9-469 18842156 PMC2576258

[B29] Wawrzynski A Ashfield T Chen NW Mammadov J Nguyen A Podicheti R Cannon SB Thareau V Ameline-Torregrosa C Cannon E Chacko B Couloux A Dalwani A Denny R Deshpande S Egan AN Glover N Howell S Ilut D Lai H Del Campo SM Metcalf M O'Bleness M Pfeil BE Ratnaparkhe MB Samain S Sanders I Ségurens B Sévignac M Sherman-Broyles S Tucker DM Yi J Doyle JJ Geffroy V Roe BA Maroof MA Young ND Innes RW Replication of nonautonomous retroelements in soybean appears to be both recent and common Plant Physiol 2008 148 4 1760 71 10.1104/pp.108.127910 18952860 PMC2593652

[B30] Vitte C Panaud O LTR retrotransposons and flowering plant genome size: emergence of the increase/decrease model Genome Res 2005 110 1-4 91 107 10.1159/000084941 16093661

[B31] Wang H Moore MJ Soltis PS Bell CD Brockington SF Alexandre R Davis CC Latvis M Manchester SR Soltis DE Rosid radiation and the rapid rise of angiosperm-dominated forests Proc Natl Acad Sci USA 2009 106 10 3853 8 10.1073/pnas.0813376106 19223592 PMC2644257

[B32] Wikström N Savolainen V Chase MW Evolution of the angiosperms: calibrating the family tree Proc Biol Sci 2001 268 1482 2211 20 11674868 10.1098/rspb.2001.1782 PMC1088868

[B33] Magallón S Castillo A Angiosperm diversification through time American Journal of Botany 2009 96 349 365 10.3732/ajb.0800060 21628193

[B34] Costa J de Melo D Gouveia Z Cardoso H Peixe A Arnholdt-Schmitt B The alternative oxidase family of Vitis vinifera reveals an attractive model to study the importance of genomic design Physiol Plant 2009 137 4 553 65 10.1111/j.1399-3054.2009.01267.x 19682279

[B35] Tsukahara S Kobayashi A Kawabe A Mathieu O Miura A Kakutani T Bursts of retrotransposition reproduced in Arabidopsis Nature 2009 461 7262 423 6 10.1038/nature08351 19734880

[B36] Wessler S Bureau T Whitea S LTR-retrotransposons and MITEs: important players in the evolution of plant genomes Curr Opin Genet Dev 1995 5 6 814 21 10.1016/0959-437X(95)80016-X 8745082

[B37] Hawkins J Proulx S Rapp R Wendel J Rapid DNA loss as a counterbalance to genome expansion through retrotransposon proliferation in plants Proc Natl Acad Sci USA 2009 106 42 17811 6 10.1073/pnas.0904339106 19815511 PMC2764891

[B38] Barnaud A Laucou V This P Lacombe T Doligez A Linkage disequilibrium in wild French grapevine, Vitis vinifera L. subsp. silvestris Heredity 2009 in press 19844269 10.1038/hdy.2009.143

[B39] Baucom R Estill J Leebens-Mack J Bennetzen J Natural selection on gene function drives the evolution of LTR retrotransposon families in the rice genome Genome Res 2009 19 2 243 54 10.1101/gr.083360.108 19029538 PMC2652206

[B40] Naito K Cho E Yang G Campbell M Yano K Okumoto Y Tanisaka T Wessler S Dramatic amplification of a rice transposable element during recent domestication Proc Natl Acad Sci USA 2006 103 47 17620 5 10.1073/pnas.0605421103 17101970 PMC1693796

[B41] Gao L McCarthy E Ganko E McDonald J Evolutionary history of Oryza sativa LTR retrotransposons: a preliminary survey of the rice genome sequences BMC Genomics 2004 5 1 18 10.1186/1471-2164-5-18 15040813 PMC373447

[B42] Parkinson J Anthony A Wasmuth J Schmid R Hedley A Blaxter M PartiGene--constructing partial genomes Bioinformatics 2004 20 9 1398 404 10.1093/bioinformatics/bth101 14988115

[B43] Parkinson J Guiliano D Blaxter M Making sense of EST sequences by CLOBBing them BMC Bioinformatics 2002 3 31 10.1186/1471-2105-3-31 12398795 PMC137596

[B44] Pertea G Huang X Liang F Antonescu V Sultana R Karamycheva S Lee Y White J Cheung F Parvizi B Tsai J Quackenbush J TIGR Gene Indices clustering tools (TGICL): a software system for fast clustering of large EST datasets Bioinformatics 2003 19 5 651 2 10.1093/bioinformatics/btg034 12651724

[B45] Altschul S Madden T Schaffer A Zhang J Zhang Z Miller W Lipman D Gapped BLAST and PSI-BLAST: a new generation of protein database search programs Nucleic Acids Res 1997 25 3389 3402 10.1093/nar/25.17.3389 9254694 PMC146917

[B46] de la Bastide M McCombie W Assembling genomic DNA sequences with PHRAP Curr Protoc Bioinformatics 2007 Chapter 11 Unit11.4 18428783 10.1002/0471250953.bi1104s17

[B47] Iseli C Jongeneel C Bucher P ESTScan: a program for detecting, evaluating, and reconstructing potential coding regions in EST sequences Proc Int Conf Intell Syst Mol Biol 1999 1999 138 48 10786296

[B48] Kent W BLAT--the BLAST-like alignment tool Genome Res 2002 12 4 656 64 11932250 10.1101/gr.229202 PMC187518

[B49] Finn RD Mistry J Tate J Coggill P Heger A Pollington JE Gavin OL Gunesekaran P Ceric G Forslund K Holm L Sonnhammer EL Eddy SR Bateman A The Pfam protein families database Nucleic Acids Research 2010 38 Database D211 222 10.1093/nar/gkp985 19920124 PMC2808889

[B50] Smit A Hubley R Green P RepeatMasker 2009 http://repeatmasker.org

[B51] Xu Z Wang H LTR_FINDER: an efficient tool for the prediction of full-length LTR retrotransposons Nucleic Acids Res 2007 35 Web Server W265 8 10.1093/nar/gkm286 17485477 PMC1933203

[B52] SanMiguel P Gaut B Tikhonov A Nakajima Y Bennetzen J The paleontology of intergene retrotransposons of maize Nature 1998 20 43 45 10.1038/1695 9731528

[B53] Jukes T Cantor C Munro H Evolution of protein molecules 1990 Mammalian protein metabolism. Academic press 21 132

[B54] R Development Core Team R: A language and environment for statistical computing 2009 R Foundation for Statistical Computing, Vienna, Austria http://www.R-project.org ISBN 3-900051-07-0

